# Development of AI-driven prediction models to realize real-time tumor tracking during radiotherapy

**DOI:** 10.1186/s13014-022-02012-7

**Published:** 2022-02-23

**Authors:** Dejun Zhou, Mitsuhiro Nakamura, Nobutaka Mukumoto, Hiroaki Tanabe, Yusuke Iizuka, Michio Yoshimura, Masaki Kokubo, Yukinori Matsuo, Takashi Mizowaki

**Affiliations:** 1 Division of Medical Physics, Department of Information Technology and Medical Engineering, Human Health Sciences, Graduate School of Medicine, Kyoto University, 53 Kawahara-Cho, Shogoin, Sakyo-ku, Kyoto, 606-8507 Japan; 2 Department of Radiation Oncology and Image-Applied Therapy, Graduate School of Medicine, Kyoto University, Kyoto, Japan; 3 Department of Radiological Technology, Kobe City Medical Center General Hospital, Hyogo, Japan; 4 Department of Radiation Oncology, Kobe City Medical Center General Hospital, Hyogo, Japan

**Keywords:** Real-time tumor tracking, Tumor motion prediction, Convolutional neural network, Adaptive neuro-fuzzy inference system

## Abstract

**Background:**

In infrared reflective (IR) marker-based hybrid real-time tumor tracking (RTTT), the internal target position is predicted with the positions of IR markers attached on the patient’s body surface using a prediction model. In this work, we developed two artificial intelligence (AI)-driven prediction models to improve RTTT radiotherapy, namely, a convolutional neural network (CNN) and an adaptive neuro-fuzzy inference system (ANFIS) model. The models aim to improve the accuracy in predicting three-dimensional tumor motion.

**Methods:**

From patients whose respiration-induced motion of the tumor, indicated by the fiducial markers, exceeded 8 mm, 1079 logfiles of IR marker-based hybrid RTTT (IR Tracking) with the gimbal-head radiotherapy system were acquired and randomly divided into two datasets. All the included patients were breathing freely with more than four external IR markers. The historical dataset for the CNN model contained 1003 logfiles, while the remaining 76 logfiles complemented the evaluation dataset. The logfiles recorded the external IR marker positions at a frequency of 60 Hz and fiducial markers as surrogates for the detected target positions every 80–640 ms for 20–40 s. For each logfile in the evaluation dataset, the prediction models were trained based on the data in the first three quarters of the recording period. In the last quarter, the performance of the patient-specific prediction models was tested and evaluated. The overall performance of the AI-driven prediction models was ranked by the percentage of predicted target position within 2 mm of the detected target position. Moreover, the performance of the AI-driven models was compared to a regression prediction model currently implemented in gimbal-head radiotherapy systems.

**Results:**

The percentage of the predicted target position within 2 mm of the detected target position was 95.1%, 92.6% and 85.6% for the CNN, ANFIS, and regression model, respectively. In the evaluation dataset, the CNN, ANFIS, and regression model performed best in 43, 28 and 5 logfiles, respectively.

**Conclusions:**

The proposed AI-driven prediction models outperformed the regression prediction model, and the overall performance of the CNN model was slightly better than that of the ANFIS model on the evaluation dataset.

## Background

During beam delivery, the targets—particularly those located in the thoracic and abdominal regions—move during respiration [1]. Conventionally, the internal target volume method is the most common approach to perform radiation therapy for such targets, as it sufficiently covers the range of movement [2]. However, in this approach, along with the target, the surrounding normal tissue is also irradiated at a high dose, which may have adverse consequences.

With recent advances in radiotherapy systems, four-dimensional (4D) radiotherapy can be performed in clinical practice. In this approach, breath-hold, respiratory gating, and real-time tumor tracking (RTTT) techniques can be adopted to reduce the effects of respiratory motion [3]. In particular, through the RTTT, the beam position can be changed with the target position, thereby minimizing the occurrence of the adverse events caused by the motion of the target without burdening the patient's breath or extending the treatment time [4].

In September 2011, we started infrared reflective (IR) marker-based RTTT (IR Tracking) with a gimbal-head radiotherapy system, known as Vero4DRT (Hitachi Ltd., Tokyo, Japan, and Brainlab AG, Feldkirchen, Germany) [5], for lung [6], liver [7], and pancreatic [8] cancer patients. IR Tracking is a hybrid RTTT technique that combines direct positioning and indirect RTTT methods [9]. The IR Tracking method predicts the internal target position with the positions of IR markers placed on the patient’s abdominal wall by using the regression-based prediction model. The prediction accuracy of IR Tracking depends considerably on the performance of the prediction model [10]. We have observed that the regression-based prediction model does not represent the tumor motion accurately. In this regard, the existing regression-based prediction model implemented in Vero4DRT can be improved in terms of accuracy [11–13].

Artificial intelligence (AI) techniques are being extensively and rapidly implemented in radiotherapy [14]. In general, support vector regression [15, 16], Gaussian process regression [17], neural networks [18, 19], and fuzzy logic [20, 21] can be applied to predict target positions with external surrogate positions; however, these algorithms are associated with specific limitations when applied to prediction models. The performance of the support vector regression is not satisfactory when data is used from free-breathing humans [16]. Moreover, the Gaussian process regression approach was tested only on a respiratory simulation phantom model with a rubber hot-water bottle [17]. Consequently, the simulation cannot accurately reflect the actual patient breathing and tumor motion. Although neural networks and fuzzy logic exhibit a satisfactory performance, in the research conducted with the use of these techniques, only 3 and 20 patients were tested, respectively [18, 20, 21]. In the work presented by Teo et al., only the tumor motion in the superior–inferior direction was predicted with the input of internal target position with an electronic portal imaging device (EPID) at the frequency at 7.5 Hz [19, 22]. In addition, their approach cannot be performed if the internal target positions are invisible on EPID. Thus, the approach was rendered unsuitable for intensity-modulated radiation therapy or volumetric modulated arc therapy.

Considering such aspects, two AI-driven prediction models, expected to have enhanced accuracy, were developed in this work. A convolutional neural network (CNN)-driven model with fine-tuning, and a model driven by an adaptive neuro-fuzzy inference system (ANFIS) with a pattern search algorithm were used. Compared to the regression-based prediction model, the AI-driven prediction models can better predict the internal target position in three dimensions (3D) using the external marker position without changes in the current workflow. Compared to the CNN model, the ANFIS model contained fewer layers, does not require building a reference model, and does not need too much data for training. With two different approaches based on AI, it may be possible to have more alternatives for future research. By comparing and analyzing the performance of the different prediction models in the same scenarios, we can obtain a better understanding of their characteristics. The performance of the proposed prediction models was further compared to that of the regression model and was evaluated to enhance the understanding of the difference between the AI mechanisms and the conventional approach involved in developing prediction models.

## Methods

### IR tracking procedure of Vero4DRT

The details of the RTTT procedure for Vero4DRT were described in a previous study [10].

Before the treatment beam delivery, an IR camera mounted on the ceiling of the treatment room monitors the motion of the one-dimensional (1D) IR markers placed on the abdominal wall every 16.7 ms. In addition, the orthogonal kV X-ray imaging subsystem implemented in Vero4DRT detects the fiducial markers as surrogates for the detected target positions (*P*_detect_) every 80–640 ms. These motions are monitored for 20–40 s. After monitoring, a regression-based prediction model $f\left( {P_{{{\text{IR}}}} ,v_{{{\text{IR}}}} } \right)$ is built as follows:

1 $$f\left( {P_{{{\text{IR}}}} ,v_{{{\text{IR}}}} } \right) = aP_{{{\text{IR}}}}^{2} + bP_{{{\text{IR}}}} + c + dv_{{{\text{IR}}}}^{2} + ev_{{{\text{IR}}}} ,$$

where *P*_IR_ is the averaged 1D IR marker position of multiple IR markers’ measurements, $v_{{{\text{IR}}}}^{{}}$ is the averaged vertical velocity of the IR markers. The positions of the IR markers are predicted from the past position (25 ms before) to compensate for the system delay. The parameters from *a* to *e* are optimized by linear regression.

During the treatment beam delivery, the future 3D target position (*P*_predict_) is predicted from the position and velocity of IR markers with the use of Eq. (1). Additionally, the internal target position is monitored every 1 s to verify the results produced by the prediction model.

### Data characteristics

This research was performed in accordance with the Declaration of Helsinki and was approved by the institutional review board. A total of 1079 logfiles were extracted from Vero4DRT. These logfiles were obtained from lung, liver, and pancreatic cancer patients whose 3D respiration-induced motion of the tumor, as indicated by the fiducial markers, exceeded 8 mm during IR Tracking. We selected 8 mm based on previous studies, considering the adaptation of respiratory motion management techniques [23] and significance of long- and short-term tumor motion variability [24]. All the patients were breathing freely, and more than four external IR markers were attached on the abdominal walls of each patient. The logfiles were acquired when building the prediction model. In our clinical practice protocol, we recorded IR marker positions during an interval of 20–40 s at a frequency of 60 Hz before treatment beam delivery and detected target positions indicated by the implanted markers for the same period at intervals ranging from 80 to 640 ms. During this period, the regression prediction model was constructed [10–12].

The 1079 logfiles were randomly divided into two datasets. To improve the prediction accuracy, as many datasets as possible are required. In this study, the historical dataset for the CNN model contained 1003 logfiles, and the remaining 76 logfiles complemented the evaluation dataset. The evaluation dataset was used to evaluate the performance of the prediction models. For each logfile in the evaluation dataset, the first three quarters were used as the training periods for transfer learning to build the patient-specific prediction models. The last quarter was the testing period and was used to test the performance of the prediction model.

Table 1 summarizes the IR marker motion patterns for the logfiles in the evaluation dataset. The mean and standard deviation (SD) values of the peak-to-peak motion range (*R*), the breathing period (*T*), and the 90th percentile of the respiratory velocity (*v*_90_) during the training and testing periods were calculated separately. The absolute difference of each value was calculated to show whether the respiratory motion was smooth and stable. Table 2 shows the summary of tumor motion range in three directions. The mean and SD values of the detected target motion ranges in the right–left, superior–inferior, and anterior–posterior directions during the training and testing period were calculated.

**Table 1:** Summary of infrared reflective (IR) marker motion characteristics for evaluation dataset

	Training period	Testing period	Absolute difference	*p*-value
*R* (mm)	7.2 ± 2.9 [2.9–15.5]	7.1 ± 3.0 [2.9–16.5]	1.2 ± 1.7 [0.0–10.4]	0.87
*T* (s)	4.2 ± 1.5 [2.5–8.2]	4.2 ± 1.4 [1.1–8.4]	0.6 ± 0.9 [0.0–5.7]	0.97
*v*_90_ (mm/s)	7.3 ± 2.0 [4.0–13.5]	7.4 ± 2.2 [3.8–17.5]	0.8 ± 0.9 [0.0–5.0]	0.74

*R*, Peak-to-peak motion range; *T*, period; *v*_90_, 90th percentile of respiratory velocity. Values are presented as means ± standard deviations (SD) [range, min–max]. *P*-values are the paired *t*-test results between training period and testing period

**Table 2:** Summary of detected target motion ranges in three directions for the evaluation dataset

	Training period	Testing period	Absolute difference	*p*-value
Right–left (mm)	2.1 ± 1.7 [0.5–9.1]	2.1 ± 1.9 [0.4–10.8]	0.4 ± 0.4 [0.0–2.2]	0.76
Superior–inferior (mm)	16.4 ± 8.1 [7.6–37.1]	16.5 ± 8.6 [7.4–46.0]	2.4 ± 3.8 [0.0–27.1]	0.92
Anterior–posterior (mm)	3.0 ± 1.3 [0.8–6.4]	3.1 ± 2.4 [0.8–19.9]	0.7 ± 1.9 [0.0–16.7]	0.64

Values are shown in means ± SD [range, min–max]. *P*-values are the paired *t*-test results between training period and testing period

### CNN-driven prediction model

**Fig. 1:**
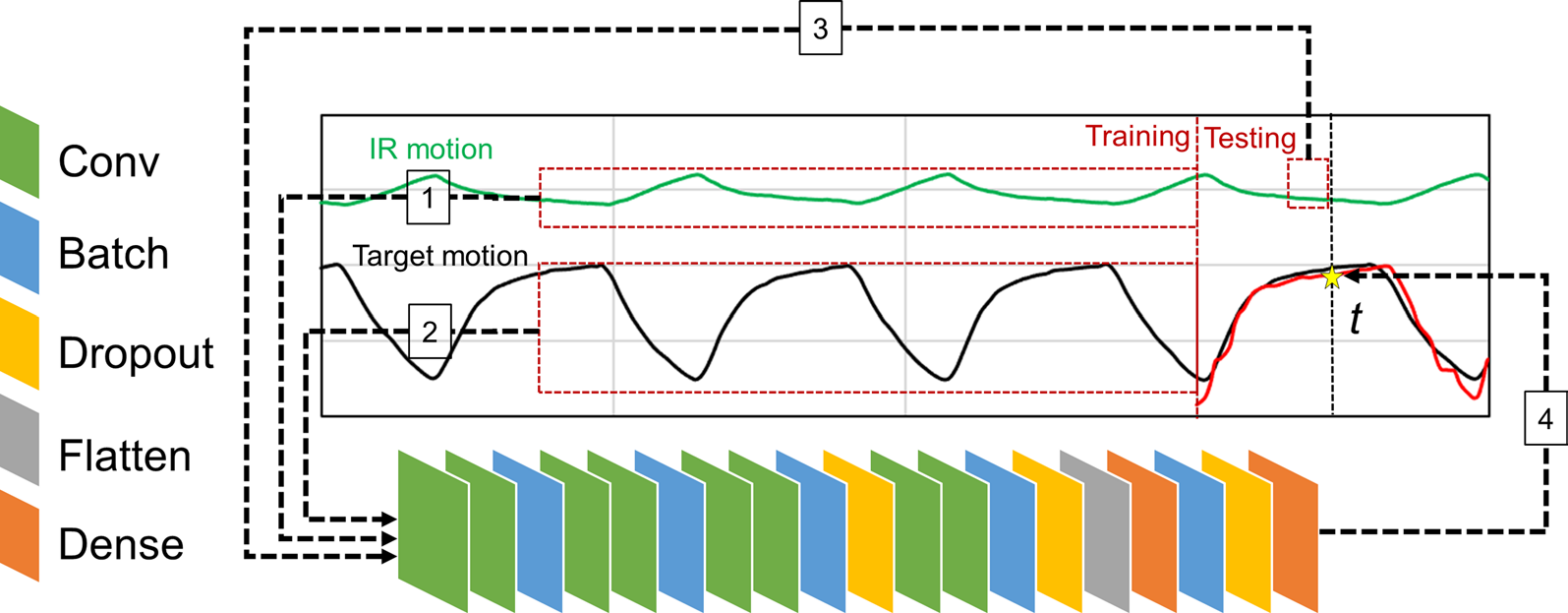
Schema of the convolutional neural network (CNN) model. The CNN model consisted of eight convolution layers (green), five batch normalization layers (blue), three dropout layers (yellow), a flatten layer (gray), and two dense layers (orange)

The schema for the CNN model is shown in Fig. 1. The CNN model was constructed to have nineteen layers in total, with eight convolution layers, five batch normalization layers, three dropout layers, a flatten layer, and two dense layers. In this study, the model was implemented in Python 3.6.4 and Keras 2.1.2. The Adam optimizer was employed, and the loss function was the mean value of the absolute differences between the detected and predicted target positions presented by the CNN model.

The CNN model consisted of training based on a large patient population and conducting patient-specific transfer learning. Initially, a single reference model was developed with the use of the historical dataset. In this process, CNN could learn and acquire knowledge from the dataset. The reference model was trained for 20 epochs with a learning rate of 0.001. The reason for the setting of parameters was based on the consideration that the reference model was fine-tuned later. The construction of the reference model involved the following steps:

1. Randomly extract the data for 12 s *P*_*IR*_ (720 positions) for IR marker No. 1 from a single historical logfile (dashed line 1 in Fig. 1).
2. Extract 25 *P*_detect_ values from the same period at equal intervals (dashed line 2 in Fig. 1).
3. Extract 50 *P*_*IR*_ immediately before time *t* in the last quarter of a single historical logfile (dashed line 3 in Fig. 1).
4. Calculate *P*_predict_ at time *t* [*P*_predict_(*t*)] in the last quarter of the single historical logfile with the data from steps 1–3 (dashed line 4 in Fig. 1).
5. Train the reference model and learn the weights based on the aforementioned steps.
6. Repeat the steps until all the IR markers, time intervals, and entire historical dataset are covered.

For each logfile in the evaluation dataset, the reference model was tuned using the data in the first three-quarters of the logfile (training period). The tuned reference model was trained for five epochs, with the learning rate ranging linearly from 0.0005 to 0.0001. For each logfile, the CNN model calculated *P*_predict_ using the following steps:

1. Extract the data for the last 12 s *P*_*IR*_ (720 positions) of IR marker No. 1 from the training period.
2. Extract 25 *P*_detect_ values from the same period with equal intervals.
3. Extract 50 *P*_*IR*_ immediately before time *t* in the training period.
4. Tune the reference model with *P*_detect_(*t*) and the data acquired in steps 1–3 until all the time points in the training period are covered.
5. Calculate *P*_predict_(*t*) one at a time. The input was 50 *P*_*IR*_ immediately before time *t*, with the last 720 *P*_*IR*_ and 25 *P*_detect_ values of the training period.
6. Repeat step 5 until all the markers are covered.
7. Calculate the average value of *P*_predict_(*t*) calculated with each IR marker. This result is the final prediction result.
8. Repeat step 7 until all the time *t* in testing period is covered.

### ANFIS-driven model

The ANFIS technique combined the adaptive neural network and fuzzy inference system (FIS). The FIS used the fuzzy set theory and fuzzy rules to map the inputs to the outputs. The fuzzy set was generated through a clustering algorithm, and the mapping was performed by considering the membership function and fuzzy rules. Subsequently, a five-layer adaptive neural network was adapted as a machine learning approach to tune the FIS parameters. The detailed information regarding ANFIS can be found in [25] and [26].

**Fig. 2:**
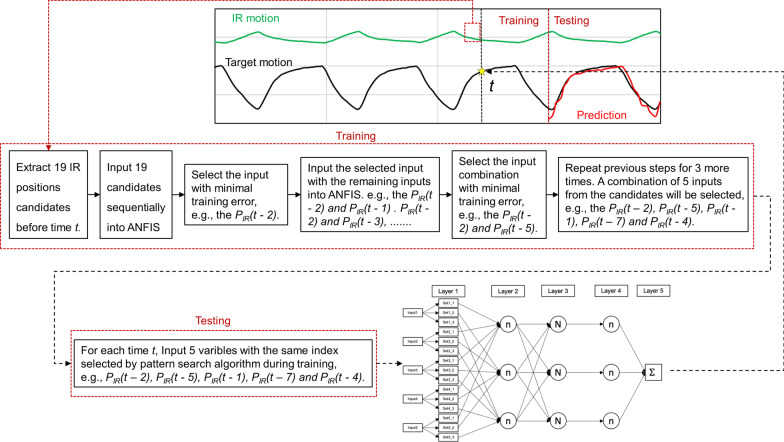
Schema of the adaptive neuro-fuzzy inference system (ANFIS) model. The node with the capitalized N letter refers to the normalized fuzzy inference system (FIS), while the node with lowercase n refers to non-normalized FIS

The schema for the ANFIS model presented in this work is shown in Fig. 2. The input of the model was the IR marker positions, and the output was the predicted target position in 3D. The ANFIS model predicted the target position one at a time, similar to the CNN and regression-based models. In this work, FIS and ANFIS were implemented in MATLAB (R2020a, MathWorks, Natick, MA, USA) using the Fuzzy Logic and ANFIS Toolboxes. The Fuzzy Logic Toolbox provided the Mamdani and Sugeno FIS types. The Sugeno-type FIS was adopted for the ANFIS model because of its higher computational efficiency compared to that of the Mamdani-type FIS. The hybrid method was selected as the optimization method in the ANFIS Toolbox. In particular, the hybrid method combined the backpropagation and least-squares estimation techniques for the parameters of the input and output membership functions, respectively. In the ANFIS model, the ANFIS was implemented with a pattern search algorithm, which can sequentially select the input data from the candidates to optimize the total squared error of the ANFIS during the training. For each logfile, the ANFIS model calculated *P*_predict_ based on the following steps:

1. Extract *P*_detect_ and *P*_*IR*_ of IR marker No. 1 from the training period of each logfile.
2. For each *P*_detect_(*t*), prepare 11 input candidates of *P*_*IR*_ (herein, values of *P*_*IR*_(*t*) to *P*_*IR*_(*t* − 10) were selected owing to their proximity to *P*_detect_(*t*)). Eight additional candidates, specifically, *P*_*IR*_(*t* − 15), *P*_*IR*_(*t* − 20), *P*_*IR*_(*t* − 25), *P*_*IR*_(*t* − 30), *P*_*IR*_(*t* − 35), *P*_*IR*_(*t* − 40), *P*_*IR*_(*t* − 45), and *P*_*IR*_(*t* − 50), were selected as they may influence *P*_detect_(*t*); the numbers refer to the index in the array of the IR marker motion data). The corresponding *P*_*detect*_ values of the 11 input candidates were not extracted.
3. Process the 19 input candidates sequentially and select the candidate with the minimum training error in the ANFIS.
4. Sequentially process the remaining input candidates with the selected candidates and repeat steps 3 and 4 until five inputs are selected from the 19 candidates. These five inputs were considered to be the most relevant patterns of *P*_*IR*_ with *P*_detect_(*t*).
5. Train the model with *P*_predict_(*t*) and the most relevant pattern of *P*_*IR*_ during the training period for four epochs.
6. Calculate *P*_predict_(*t*) with the most relevant pattern of *P*_*IR*_ during the testing period.
7. Repeat step 6 until all the IR marks are covered.
8. The average value of *P*_predict_(*t*) calculated using each IR marker is the final prediction result.
9. Repeat step 8 until all the time t in testing period is covered.

### Data analysis

The proposed prediction models processed each of the randomly selected 76 logfiles by using the aforementioned procedure. For each logfile, the CNN model fine-tuned the reference model during the training period and yielded the prediction results for the testing period. The ANFIS model used the pattern search algorithm and developed the ANFIS for each logfile during the training period and predicted the target positions during the testing period. To enable a comparison, a regression model was constructed during the training period [13], and *P*_predict_ values were calculated during the testing period for each of the 76 logfiles.

During data analysis, the detected target position was considered as the ground truth of the prediction. The overall performance of the prediction model was ranked by the percentage of *P*_predict_ within 2 mm of *P*_detect_ at each recorded time. Furthermore, the cumulative percentage curve of 3D prediction positional error for the three models was analyzed.

According to the International Organization of Standardization (ISO) standard 5725-1 [27], the accuracy of a measurement is a combination of the trueness (mean error) and precision (standard deviation of the error, SD). In this study, the performances of the proposed CNN, ANFIS, and regression model on a single logfile were also evaluated in terms of accuracy. The mean absolute error (MAE) and SD between *P*_predict_ and *P*_detect_ were calculated for each logfile from the evaluation dataset. The parametric paired *t*-test was performed to evaluate the statistical significance of MAE between the AI-driven and regression model; the level of significance was set to 0.05.

As the CNN model learnt from the historical dataset and the ANFIS model was trained and tested on a single logfile, the change in the respiratory range, period, and velocity measured by the IR markers for the training and testing periods of the logfile might have influenced the comparison of the proposed prediction models. To quantify these changes, the variables *δ*_*r*_, *δ*_*p*_, and *δ*_*v*,_ that indicated the degrees of change in the respiration range, period, velocity between the training and testing periods, respectively, were defined and calculated for each logfile:

2 $$\delta = \left| {\frac{{Value_{test} }}{{Value_{train} }} - 1} \right|,$$

where *Value*_*test*_ and *Value*_*train*_ represent the corresponding values during the testing and training periods, respectively. For the range and period, the values were the mean range and period, respectively. For velocity, the values were the 90th percentiles of the IR velocity during the testing period and training period, respectively. A larger *δ* value indicates a greater change. In particular, for stable respiratory patterns, *δ* will be close to zero.

## Results

The averaged training times of the CNN and ANFIS models for each logfile were approximately 12 s and 95 s, respectively.

**Fig. 3:**
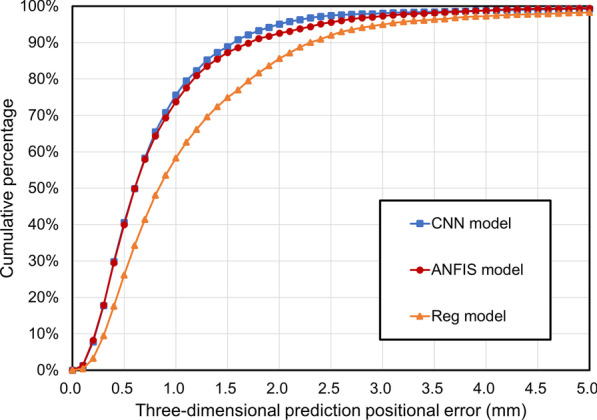
Cumulative percentage curve regarding *P*_predict_ within *P*_detect_ in designated distance

The cumulative percentage curve of the 3D prediction positional error is also consistent with the aforementioned result (Fig. 3). As shown in Fig. 3, the CNN and ANFIS models exhibit nearly the same cumulative percentage distribution when the 3D prediction positional error is smaller than 1 mm. When the distance between *P*_predict_ and *P*_detect_ ranged from 1 to 3 mm, the CNN model exhibited the highest performance. Overall, the performance of the AI-driven models was better than that of the regression model. The percentages of 3D prediction positional error within 2 mm were 95.1%, 92.6% and 85.6% for the CNN, ANFIS, and regression models, respectively. This indicates that the CNN model showed the best performance among the three models. There were significant differences in MAE between the CNN and regression model (*p* < 0.05) and between the ANFIS and regression model (*p* < 0.05).

**Fig. 4:**
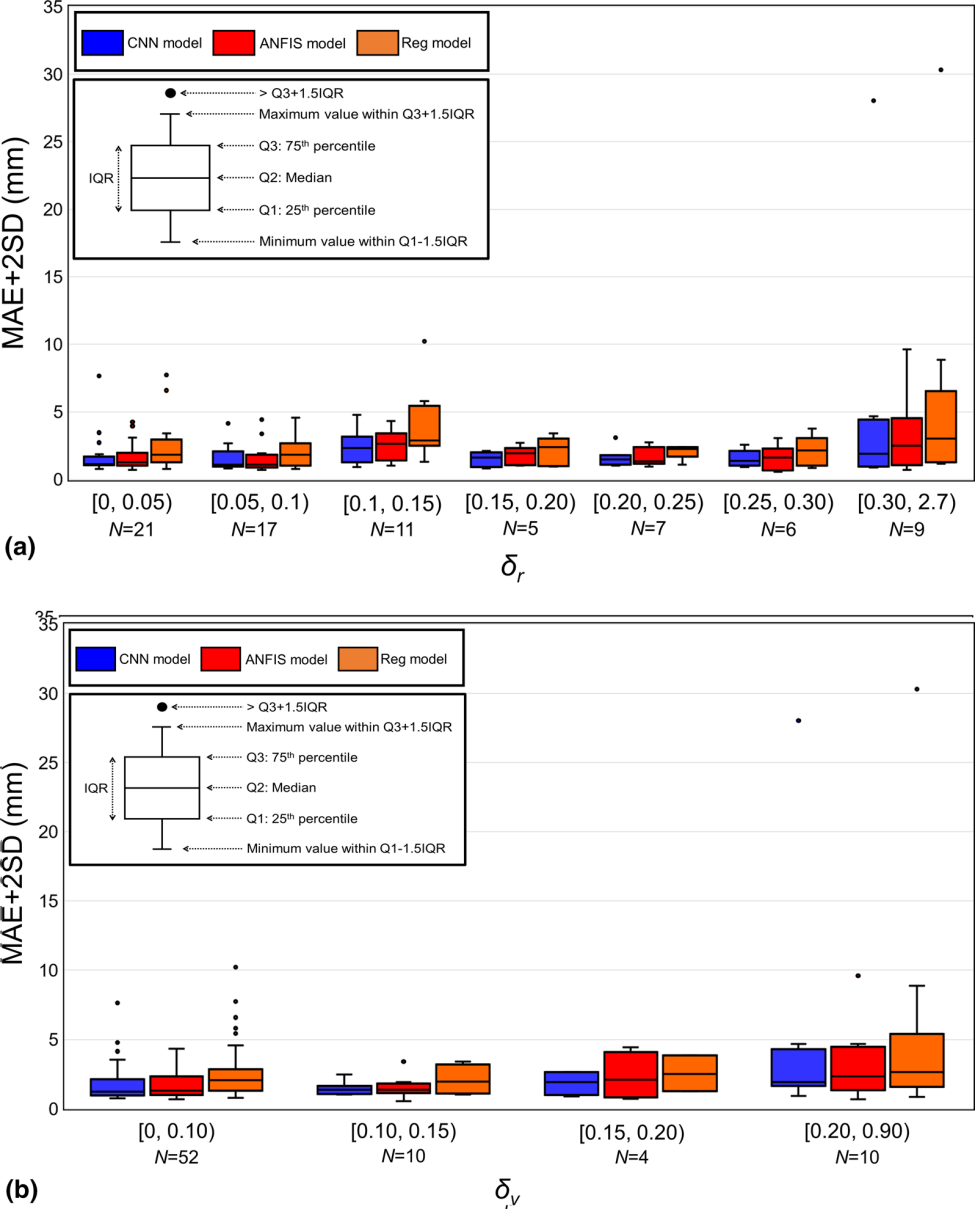

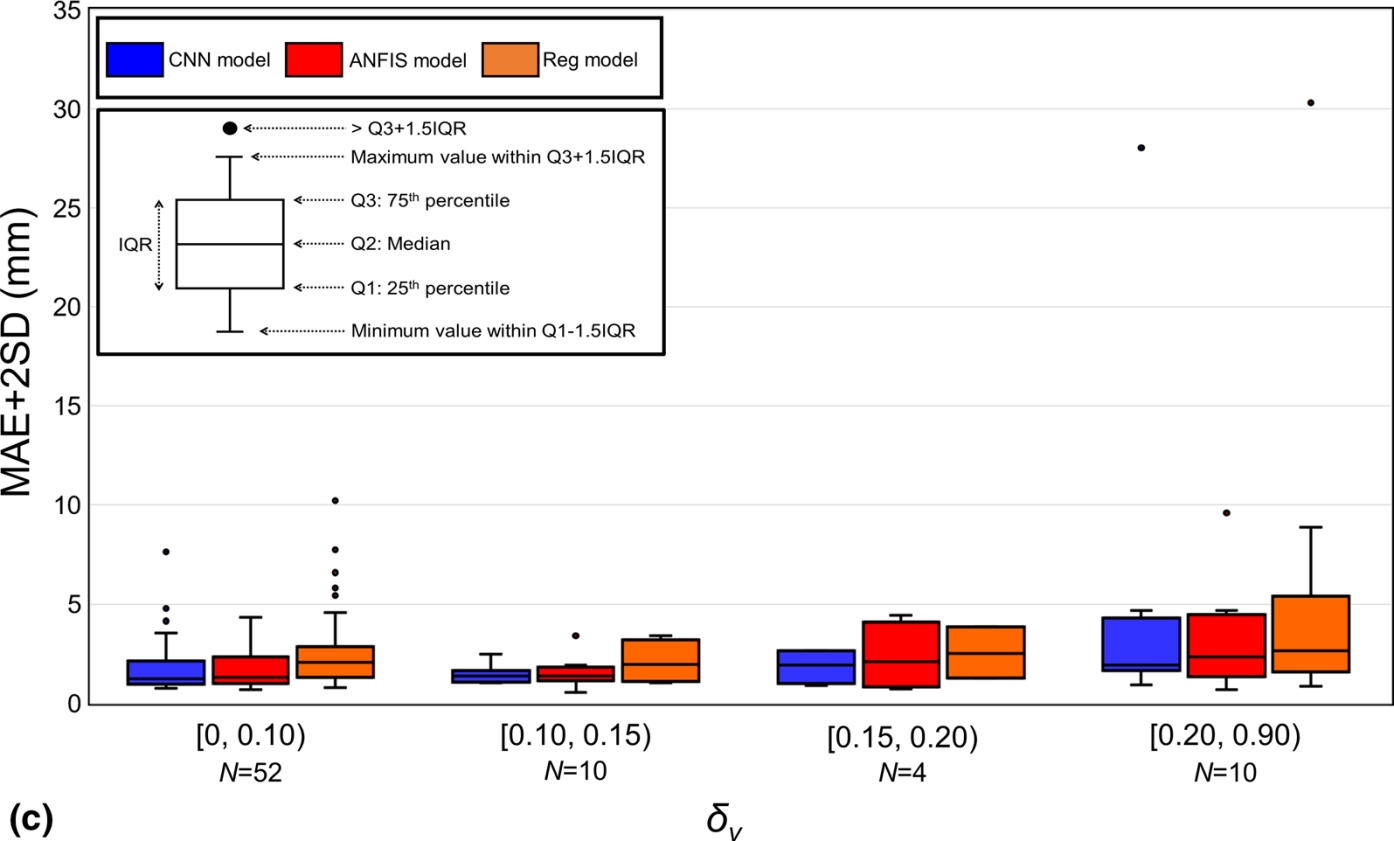
Mean absolute error (MAE) + 2 standard deviations (SD) between *P*_predict_ and *P*_detect_ versus** (a)** *δ*_*r*_,** (b)** *δ*_*p*_, and** (c)** *δ*_*v*_. The boxes represent the interquartile ranges (IQRs). Outliers were above the third quartile plus 1.5 × IQR. Blue, red, and orange represent the CNN, ANFIS, and regression models, respectively. *N* in the horizon axis refers to the number of logfile regarding to each range

The mean ± SD values of the degrees of change in the respiration range (*δ*_*r*_), period (*δ*_*p*_), and velocity (*δ*_*v*_) between the training and testing periods were 0.17 ± 0.32 (range, 0.00–2.69), 0.13 ± 0.17 (range, 0.00–0.98), and 0.10 ± 0.12 (range, 0.00–2.69), respectively. Figure 4 shows the relationships between *δ*_*r*_, *δ*_*p*_, and *δ*_*v*_, and MAE + 2SD. Figure 4a shows that the performances of the ANFIS and CNN models are comparable. Figure 4b and c show that the performance comparison of the CNN and ANFIS models is stable at all ranges for *δ*_*p*_ and *δ*_*v*_. Meanwhile, the AI-driven models always performed better than the regression model. In the following section, the performance comparison of the prediction models is discussed based on *δ*_*r*_.

**Fig. 5:**
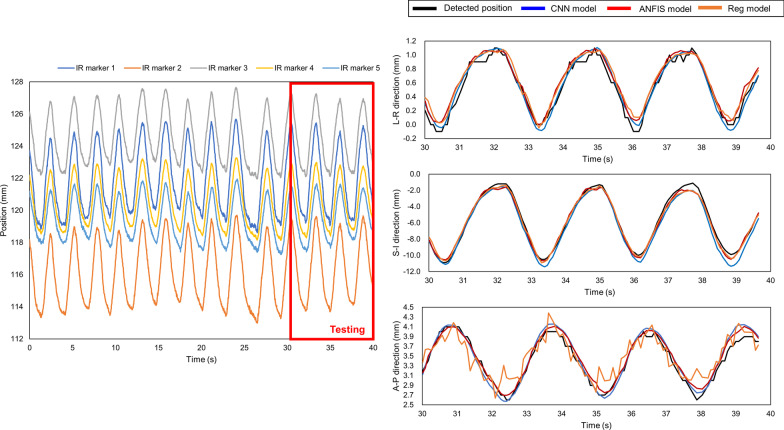
Time series data for IR marker (left side), detected and predicted target position (right side) of logfile No. 38 with a *δ*_*r*_ of 0.06. The three groups of waves on the right, from top to bottom, show the target positions in the LR, SI, and AP directions, respectively. This is a typical scenario that indicates that the respiratory was stable and the ANFIS model outperformed. The detected and predicted tumor trajectories corresponding to the testing period of the logfile, and the models give prediction results in each direction separately

**Fig. 6:**
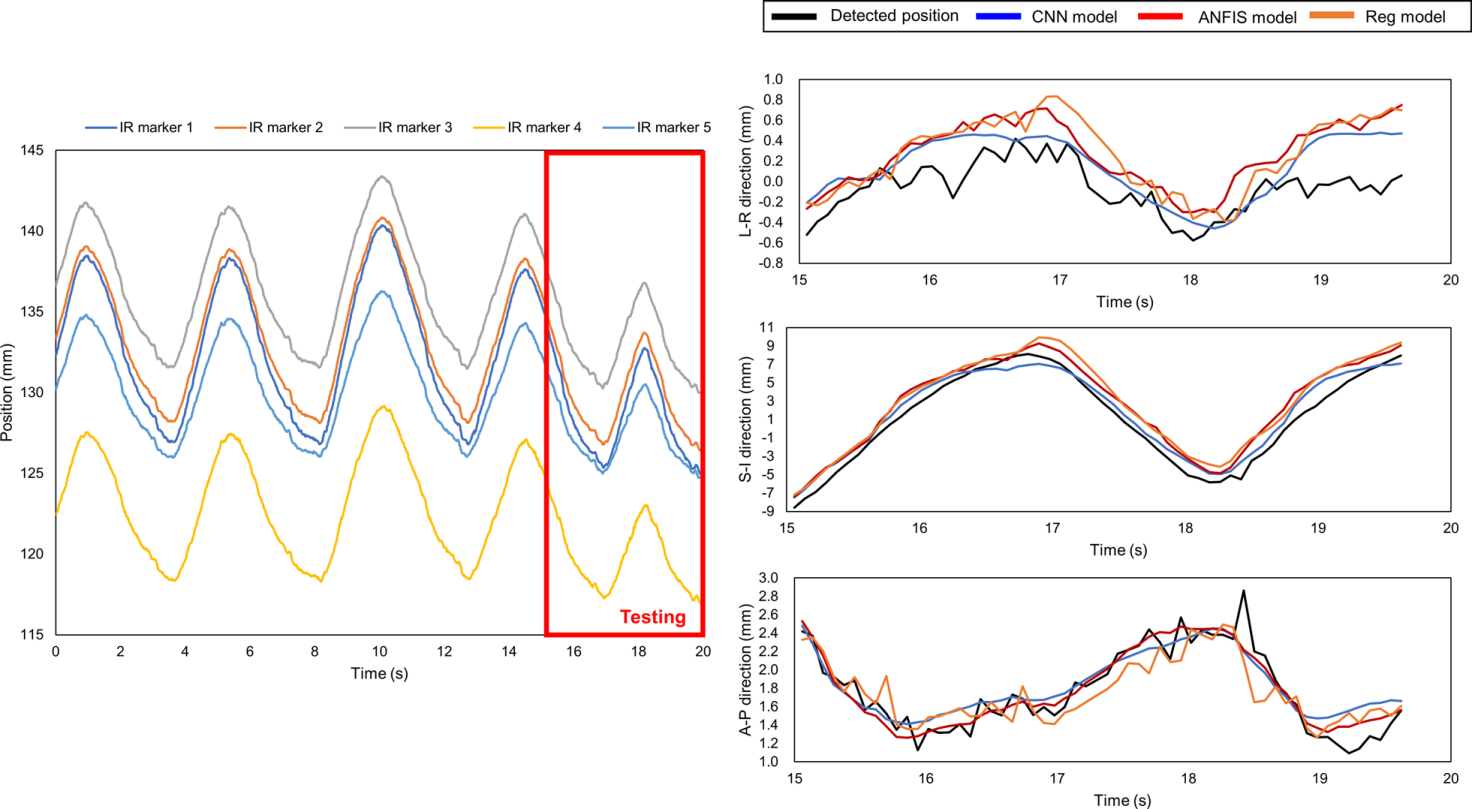
Time series data for IR marker (left side), and detected and predicted target positions (right side) of logfile No. 62 with a *δ*_*r*_ value equal to 0.36. The three groups of waves on the right, from top to bottom, show the target positions in the LR, SI, and AP directions, respectively. This is a typical scenario whereby the change in respiratory range was significant and CNN model outperformed. The detected and predicted tumor trajectories corresponding to the testing period of the logfile, and the models give prediction results in each direction separately

**Fig. 7:**
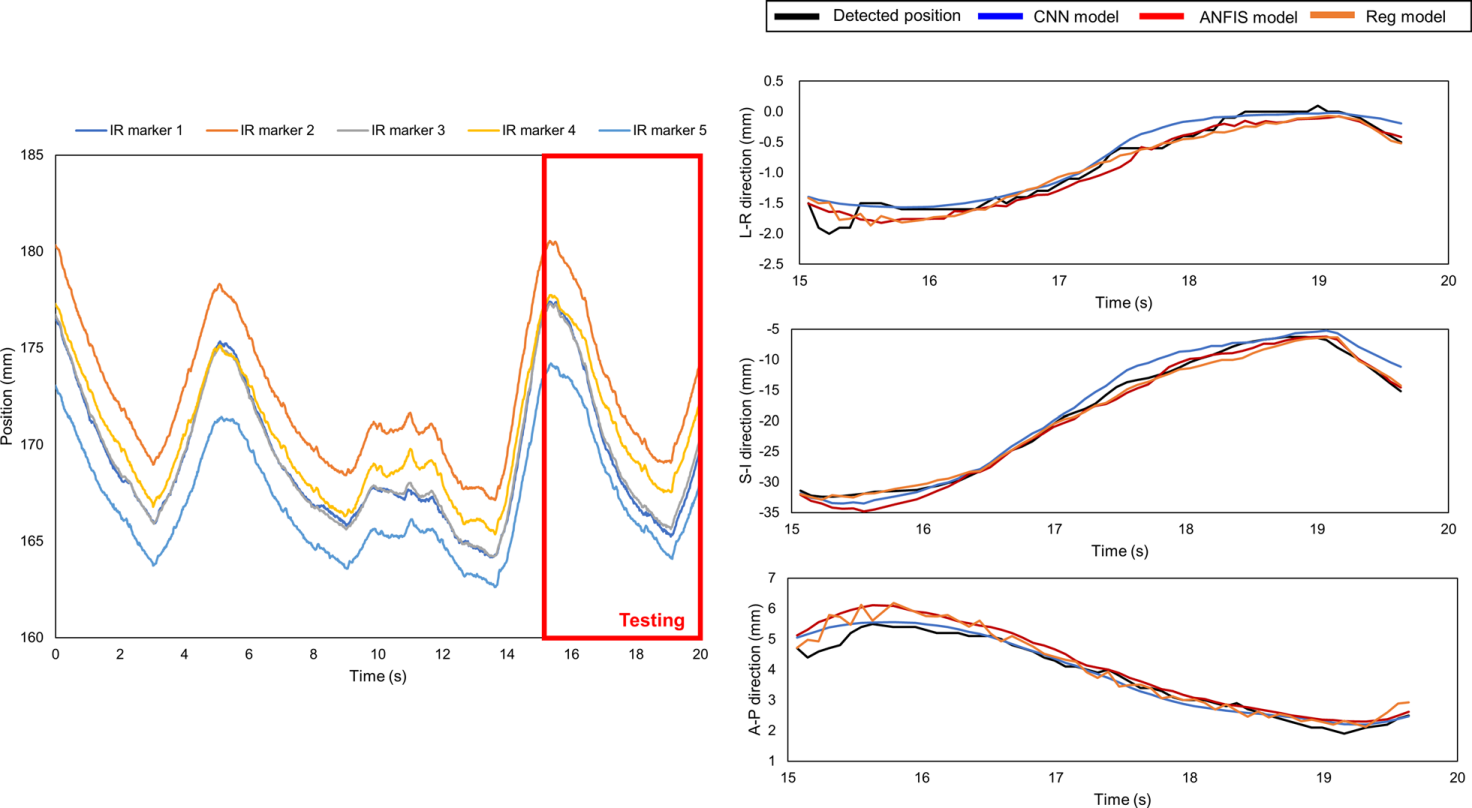
Time series data for IR marker (left side), and detected and predicted target positions (right side) of log file No. 5 with quasilinear inhale and exhale motions. The three groups of waves on the right, from top to bottom, show the target positions in the LR, SI, and AP directions, respectively. In this scenario, the inhale and exhale motions were quasilinear, and the regression model yielded the best performance. The detected and predicted tumor trajectories corresponding to the testing period of the logfile, and the models give prediction results in each direction separately

**Fig. 8:**
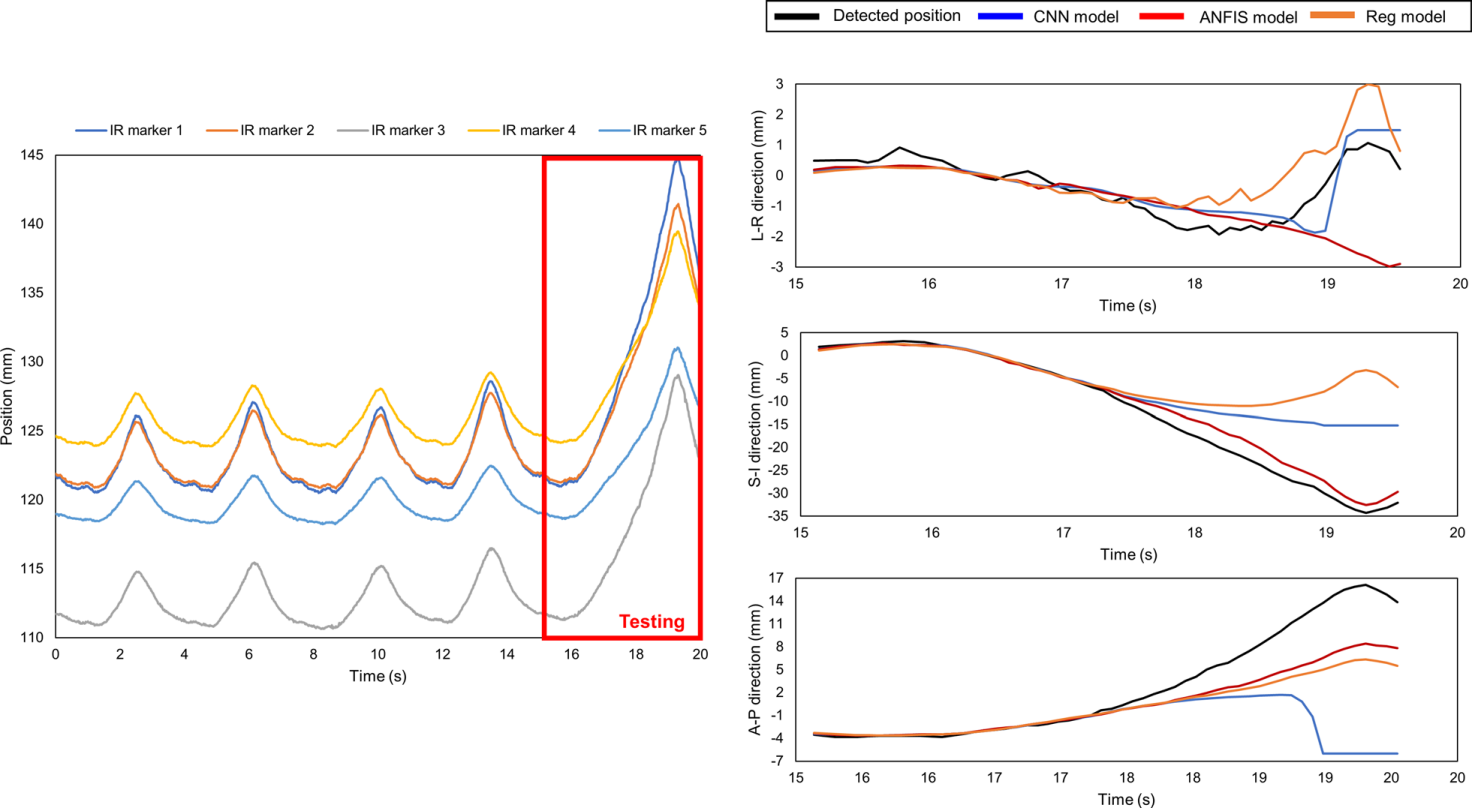
Time series data for IR marker (left side), and detected and predicted target positions (right side) of logfile No. 44 with the largest *δ*_*r*_ value equal to 2.69. The three groups of waves on the right, from top to bottom, show the target positions in the LR, SI, and AP directions, respectively. In this case, the patient inhaled deeply during the last quarter of the recording time, and none of the models could yield an acceptable prediction result. The detected and predicted tumor trajectories corresponding to the testing period of the logfile, and the models give prediction results in each direction separately

Upon comparison, it was noted that for the 43 logfiles (56.6%) showing that the CNN model outperformed the other models, the median *δ*_*r*_ value was equal to 0.12. In contrast, for the 28 logfiles wherein the ANFIS model outperformed the other models, the median *δ*_*r*_ was observed to be 0.07 (36.8%). Figure 5 shows an example of IR motion with a *δ*_*r*_ value of 0.06. The MAE + 2SD values of the CNN and ANFIS models were 1.29 and 0.71 mm, respectively. In this case, the performance of the ANFIS model was slightly better than that of the CNN model for the respiration range between the training and testing periods. As *δ*_*r*_ increased, the change in the respiration range became significant, and the CNN model outperformed the other models. For instance, in the case of logfile No. 62 with a *δ*_*r*_ value of 0.36 (Fig. 6), the MAE + 2SD values of the CNN and ANFIS models were 1.90 and 2.91 mm, respectively. Among the 76 logfiles, the regression model exhibited superior performances in the cases of five logfiles (6.6%), in which the inhale and exhale motions were quasilinear. For log file No. 5 (Fig. 7), the MAE + 2SD values of the regression, CNN, and ANFIS model were 1.87, 3.10, and 2.39 mm, respectively. Logfile No. 44 had a *δ*_*r*_ of 2.69, which corresponded to the maximum value among the 76 logfiles. As shown in Fig. 8, the patient inhales deeply during the last quarter of the recording time, leading to an irregular value of *δ*_*r*_ and produced the maximum MAE + 2SD for all three prediction models.

## Discussion

In this study, the prediction performances of the CNN and ANFIS models were compared to that of a regression model that has been utilized clinically. The CNN model was initially built as a single reference model with the historical dataset, and patient-specific transfer learning was later conducted during the training period. The ANFIS model was driven by ANFIS for each logfile, and a pattern search algorithm was adopted to select the most relevant input data. The test results showed that both AI-driven prediction models exhibited better overall performance than the regression model tested on the 76 logfiles. For each logfile, the averaged training time of the CNN and ANFIS model was approximately 12 s and 95 s, respectively. Considering the training data acquisition duration, which was 20 s to 40 s, the model construction durations of the CNN and ANFIS models were less than 52 s and 135 s, respectively. This was less than the average model construction duration of the regression model, which was 162 s, as reported by Depuydt et al. [28]. With shorter model construction durations, shorter treatment session durations can be expected if the AI-driven models are applied in clinical practice. The median value of MAE for the 76 logfiles in test dataset was 0.65, 0.66, and 1.02 mm for the CNN, ANFIS, and regression model, respectively. Thus, as the CNN and ANFIS models showed better accuracy and shorter model construction durations, less times and shorter durations of model retraining during a treatment fraction can also be expected. The durations of a treatment session will be further shorten. The *p*-values between the AI-driven models and regression model were less than 0.05, indicating that the performance of the AI-driven models was significantly better than that of the regression model. Institutionally, the setting of margin for RTTT considers both the errors induced by the internal markers [29] and the accuracy of the prediction model [10]. The implementation of AI-driven prediction models in clinical practice is expected to reduce the margin derived by the positional error of prediction models and benefit the patient in the future.

The CyberKnife (CK) system can perform RTTT using a prediction model other than Vero4DRT [9, 30]. The conventional [31] and ANFIS approach [20, 21] to construct a prediction model for a CK system was reported in previous studies. According to Poels et al. [31], the prediction accuracy of the regression model was comparable with that of conventional CK models. The results of this study demonstrated that the proposed model notably outperformed the regression model. Considering this statement, it can be concluded that the CNN and ANFIS models would outperform the conventional CK models. The work conducted by Torshabi and Ghorbanzadeh et al. adapted ANFIS to predict target motion with external marker motions for a CK system [20, 21]. In their work, the patients were divided into a control group whose tumor tracking was carried out smoothly, and a worst group that was the opposite. For their research, the average 3D root mean square error for the control group was 1.1 mm. In the present work, taking into consideration all the logfiles in the test dataset, the median value of MAE was 0.65 and 0.66 mm for the CNN and ANFIS models, respectively. This indicates that the present AI-driven models show better performances as compared to those discussed in previous works.

Compared to the Gaussian process regression model [17], which uses a rubber hot-water bottle to simulate respiratory motion, the proposed models were trained and tested with actual clinical data. The application prospects of such models in clinical practice may be more promising. Moreover, the predictions obtained using the support vector regression [16] and neural networks [18] pertained to a small patient cohort (7 and 3 patients, respectively). The present research was performed based on considerations of 76 logfiles, which corresponded to more reliable results. Comparing ours to the work done by Isaksson et al. [18], the performance of their neural network model decreased notably within 5 s; thus, the model needs to be updated within every 5 s. For the AI-driven models presented in this work, the testing period ranged from 5 to 10 s and the performance was stable, as demonstrated in Figs. 5, 6 and 7. The subsequent model accuracy will depend on *δ*_*r*_, *δ*_*p*_, and *δ*_*v*_, as shown in Fig. 4. The model presented by Teo et al. [19] required the detected target position with EPID at a frequency of 7.5 Hz and provided the prediction results only in the superior–inferior direction during the treatment beam delivery. In contrast, the target position was predicted in 3D with IR markers at 60 Hz without information of the internal target position. If the orthogonal kV X-ray imaging subsystem works at a higher frequency, the patient may receive additional dosage. Considering both our situation and the trade-off between the dosage and prediction accuracy, the models presented in this work may be more suitable for us.

Although the proposed prediction models can notably outperform the regression model, certain limitations remain. The CNN model exhibited a high performance when the scenarios were similar to those of the logfile learnt from the historical data based on the CNN. Furthermore, the ANFIS model was trained and tested solely based on the logfile and benefited from the pattern search algorithm. When the associated respiratory motion was stable, or when training was performed for a larger number of respiratory cycles, the performance of the ANFIS model would be comparable to that of the CNN model. According to this finding, the classification of the respiratory motion followed by the selection of appropriate models is expected to lead to higher prediction accuracy.

In the unique logfile No. 44 (Fig. 8a), the CNN model could not produce an accurate prediction result. This may have been caused by the imbalance in the historical dataset because the irregular respiratory patterns, for example that in logfile No. 44, were seldom included in the historical data. If additional logfiles similar to file No. 44 were to be included during the construction of the reference model, or if the irregular IR motion was included during the training period for fine-tuning, the performance of the CNN model could be improved in a similar situation. The *δ*_*r*_ value of logfile No. 44 was 2.69. It was significantly large for the ANFIS model to provide accurate results. In the case of the regression model, the velocity of the IR markers changed drastically, and the coefficients of the regression model were not suitable for this scenario; this resulted in inferior performance. Owing to the advantages of pattern recognition ability and robustness of the historical dataset, the ANFIS model corresponded to a lower MAE + 2SD (9.61 mm) in this case. In contrast, the MAE + 2SD for logfile No. 44 was 28.02 mm when the CNN model was used. Nevertheless, none of the considered prediction models could provide an acceptable prediction result (Fig. 8b). For such cases, our current clinical protocol already has a fail-safe approach. When the prediction error is larger than a predefined threshold (e.g., 3 mm, approximately half of the margin), the treatment beam will be automatically turned off [32]. When a systematic deviation is observed, the prediction model will be rebuilt and updated.

Among the 76 logfiles, the CNN, ANFIS, and regression models exhibited superior performances with minimum MAE + 2SD in 56.6%, 36.8%, and 6.6%, respectively. Even though the CNN and ANFIS models outperformed the regression model, all the possible scenarios in clinical practice cannot be covered. Specifically, for scenarios wherein the respiratory range changes considerably, as that shown in Fig. 4a whereby *δ*_*r*_ was greater than 0.3, the performances of the prediction models decreased. Based on the current study, the performance of the prediction model will decrease when *δ*_*r*_ increases. Currently, the input of the AI-driven models was the 1D IR marker position. Correspondingly, whether the relationship between the IR marker and internal target positions was stable may have a dominant influence on the performance of AI-driven prediction models. This implies that regardless of how the velocity and period changed, if the relationship of the internal and external position was stable, the performance of the prediction model was stable. However, if the respiratory range changes significantly during the testing period compared to that during the training period, the AI-driven models cannot learn the position relationship during the training period. This change would cause a negative influence on the prediction accuracy (Fig. 4a). To address such situations, the automatic beam-off function can be implemented, in which the MV beam delivery is automatically turned off if the detected 3D target position is beyond a predefined threshold [32]. In addition, the use of high-dose-rate, flattening, filter-free beams could significantly reduce the radiation delivery time, potentially contributing toward stabilization of the prediction accuracy.

Overall, this study was associated with three notable limitations. Firstly, point-by-point predictions were only performed during the last quarter of the logfiles. This corresponded to approximately 5 to 10 s of the target motion. As reported by Poels et al. [33], if a patient’s breathing motion is not stable and the prediction accuracy becomes unacceptable during treatment, the prediction model must be updated. To overcome this limitation, sequential prediction model updates can be implemented during beam delivery, or the technique presented by Teo et al. can be adapted to reduce the tracking drift in position [34]. Secondly, only the data from Vero4DRT were adapted to train and test the prediction models. The performances of the models on other systems must be examined in the future at different sampling rates, such as the CK system. Thirdly, only *δ*_*r*_, *δ*_*p*_ and *δ*_*v*_, which represented respiratory pattern changes, were considered in this study; however, there may be other factors that may need to be used to reduce the tracking accuracy. Lastly, this was a retrospective study. Currently, the models were developed, trained, and tested on previously acquired logfiles. In the future, more well-conceived experiments will be considered. The future experiments may contain longer recording durations and more irregular respiratory patterns to further improve the AI-driven models.

## Conclusions

The overall performance of the proposed CNN and ANFIS models were considerably better than that of the currently employed regression model. The CNN model performed slightly better than the ANFIS model based on tests conducted with the 76 randomly selected logfiles. Changes in the model performances were examined at different patient scenarios. In the case of considerable changes in the respiration range, the CNN model may exhibit the optimal performance. In contrast, in the case of stable respiratory ranges, the ANFIS model may achieve high prediction accuracy. Additional work can be performed to expand the application scenarios of the AI-driven models and conduct parameter optimization.

## Data Availability

Authors are not able to share data.

## Abbreviations

1D: One-dimensional
3D: Three-dimensional
4D: Four-dimensional
AI: Artificial intelligence
ANFIS: Adaptive neuro-fuzzy inference system
CK: CyberKnife
CNN: Convolutional neural network
EPID: Electronic portal imaging device
FIS: Fuzzy inference system
IR: Infrared reflective
IR Tracking: Infrared reflective marker-based hybrid real-time tumor tracking
ISO: International Organization of Standardization
MAE: Mean absolute error
*P*_detect_: Detected 3D internal position
*P*_IR_: 1D infrared reflective marker position
*P*_predict_: 3D predicted target position
*R*: Peak-to-peak motion range of infrared reflective marker
RTTT: Real-time tumor tracking
SD: Standard deviation
*T*: Breathing period
*v*_90_: 90Th percentile of the respiratory velocity
